# Unpacking the Meaning of Closeness, Reconsidering the Concept of Impact in Suicide Exposure, and Expanding Beyond Bereavement: “Just, I Hope You Don’t Forget About Us”

**DOI:** 10.1177/00302228231196616

**Published:** 2023-08-20

**Authors:** Rebecca L. Sanford, Laura M. Frey, Neetika Thind, Brock Butcher, Myfanwy Maple

**Affiliations:** 1 School of Social Work and Human Service, 33527 Thompson Rivers University, Kamloops, BC, Canada; 2 Couple & Family Therapy Program, Kent School of Social Work & Family Science, 5170 University of Louisville, Louisville, KY, USA; 3 Faculty of Medicine and Health, 1319 University of New England, Armidale, NSW, Australia

**Keywords:** suicide death, suicide exposure, cumulative exposure, meaning making, disenfranchised grief

## Abstract

Suicide exposure research has relied on samples of treatment-seeking kin, resulting in an attachment-based model centering bereavement as the most significant form of impact and obscuring other forms of significant and life-altering impact. From a community-based sample (*N* = 3010) exposed to suicide, we examine a subset (*n* = 104) with perceived high impact from the death yet low reported closeness to the person who died and analyze qualitative comments (*n* = 50). On average and out of 5.00, participants rated closeness as 1.56 but impact of death as 4.51. We illustrate dimensions of low closeness and identify themes on the meaning of impact: impact through society and systemic circumstances, impact through history and repeated exposure, impact through other people, impact as a motivator for reflection or change, and impact through shared resonance. Participants reported impact of death as significant or devastating, yet none of their comments reflected experiences typical of bereavement.

## Background

Nearly 800,000 deaths from suicide occur globally each year (World Health Organization, 2019), resulting in countless people being exposed to suicide. Research in the past decade has advanced understanding of suicide exposure—defined as “knowing or identifying with someone who has died by suicide” (Cerel et al., 2014, p. 594, p. 594)—as a public health issue by quantifying exposure to suicide death in the general public. On the low end of the range, Andriessen et al.’s (2017) meta-analysis with 18 studies estimated past-year exposure to suicide as 4.31% and life-time prevalence of exposure to suicide at 21.83%. Nationally representative data in the US (Feigelman et al., 2018) and Australia (Maple et al., 2019) published after the meta-analysis found slightly higher rates of lifetime suicide exposure prevalence—51% and 58% respectively. Despite variation based on geographic location and sampling method, it is generally accepted that exposure to suicide death affects a large portion of the general population.

Suicide postvention research has historically drawn from treatment-seeking kin (Maple et al., 2014), which has resulted in an attachment-based model for understanding the impact of suicide exposure. The Continuum Model of suicide exposure theorized a nested model illustrating the hypothesized levels of impact following suicide exposure, with a large group, estimated to be around 135 people, exposed to each suicide death (Cerel et al., 2014, 2019). The Continuum Model suggests that those least impacted may experience no significant outcomes or only minor life disruptions, while people most impacted are bereaved either for a shorter or longer period (Cerel et al., 2014). Recognizing that those impacted includes people beyond those with kinship ties, Cerel et al. (2014) propose that emotional attachment or psychological closeness to the decedent be used as a primary factor in consideration of those deemed to be most impacted by suicide exposure (often called suicide loss survivors), rather than focusing exclusively on kinship and immediate family. Psychological closeness—defined as “feelings of attachment and perceived connection toward another person” (Gino & Galinsky, 2012, p. 16, p. 16)—is not dependent upon a kinship tie to the deceased. One study found closeness to the person who died increased odds of depression, anxiety, and posttraumatic stress disorder (PTSD) (Cerel et al., 2016), suggesting perceived closeness, rather than kinship explicitly, may mediate mental health outcomes following exposure.

Although the movement away from an exclusive focus on kinship and towards consideration of psychological closeness is important for understanding impact beyond kin relationships, the Continuum Model implies impact is synonymous with bereavement and assumes bereavement is the most significant consequence of exposure to suicide. However, these entailments raise the question of whether all those who are highly impacted would describe themselves as bereaved. Bereavement is defined as the “objective situation of having lost someone significant through death” (Stroebe et al., 2008, p. 4). Within the traditional understanding of the concept, bereavement requires the loss of a significant attachment relationship. Prior research examines impact primarily through the lens of closeness and attachment, with long-term bereavement deemed the most significant form of impact, but recent research suggests this may not illustrate the full picture. In a latent profile analysis using the full sample from which the present study is drawn, we found that while four of the five groups aligned with the Continuum Model, the fifth group was discordant with the model (Bhullar et al., 2021). The discordant group generally reported high levels of impact with relatively low levels of closeness, thus raising questions about the meaning of impact and the relationship between closeness and resulting impact.

What remains unclear is how individuals who are highly impacted by a suicide loss yet were not close to the decedent would describe their experience and the meanings associated with being highly impacted. A meaning oriented framework has been used to understand suicide bereavement, particularly among kin (Begley & Quayle, 2007; Jordan, 2001; Supiano, 2012), though it has rarely been applied in the context of suicide exposure broadly (Miklin et al., 2019). With this in mind, we hypothesised that the experiences of people highly impacted by the suicide death of someone with whom they were not close would not fit the standard definition of bereavement, and if supported, perhaps services tailored to those bereaved by suicide loss would not be appropriate given the lack of attachment. To start, more information about this unique experience is needed to better strengthen the field. Therefore, we examined the subset of participants who identified low closeness yet high levels of impact from another individual’s suicide death. In this study, we sought to explore experiences where impact presumably is not synonymous with bereavement, as closeness to the decedent was low, thus indicating that it was not an attachment relationship. This study aims to contribute to the existing literature on impact to suicide exposure by exploring the following research questions:

• What are the reasons for or meanings associated with low closeness among people highly impacted by a suicide death?
• How do people with low closeness to the person who died and high impact resulting from the death describe their experience of exposure to suicide?

## Methods

### Participants

**Figure 1.**
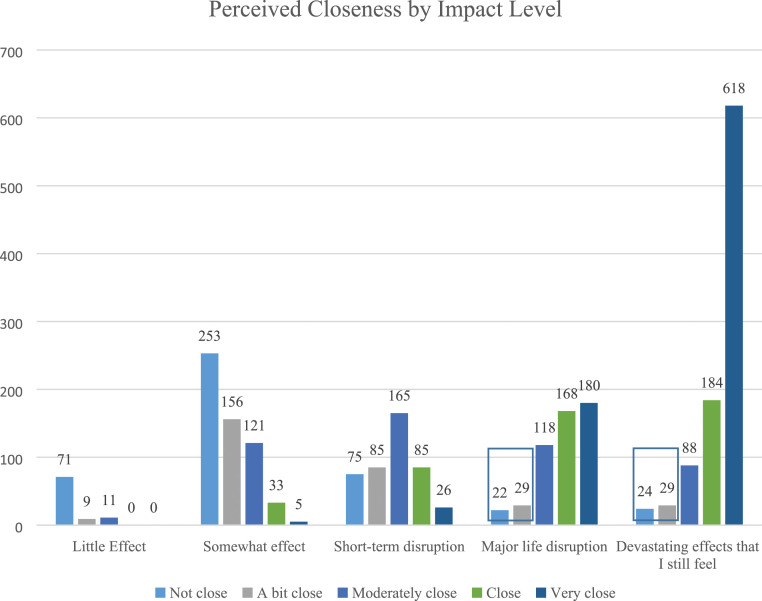
Distribution of perceived closeness by impact level. Note. Boxes indicate sample of interest (high impact/low closeness) in this study.

Ethics approval was obtained through University of New England, Australia (HE16-030). From April to August 2016, we conducted an online survey to assess exposure to suicide attempts and deaths and their subsequent impact. Distributed through Suicide Prevention Australia and other suicide prevention organizations in Australia, the survey yielded a non-representative, cross sectional, community sample of 3010 valid responses. Information about the full sample is provided in more detail in Maple & Sanford (2019). Of the full sample, 104 participants reported high impact from the death (i.e., a score of 4 or 5 on the impact item; described in Measures section below) and low closeness (i.e., a score of 1 or 2 on the closeness item). Figure 1 demonstrates the levels of impact and closeness in the full sample, with portions highlighted to indicate the subsample used in the present analyses. Some descriptive data is presented from this subsample. Of the 104 respondents indicating high impact and low closeness to the person who died from suicide, 50 provided comments when provided with an open-ended prompt. The qualitative data from these 50 participants was thematically analyzed. Participants in this sample were primarily women (80%; *n* = 40) with an average age of 46.08 (SD = 12.10; Range = 19–74). Most participants reported living in a metropolitan (60%; *n* = 30) or regional (26%; *n* = 13) location, with fewer participants living in rural (10%; *n* = 5) or remote (4%; *n* = 4) areas. Participants were primarily not of Aboriginal and Torres Strait Islander descent (90%; *n* = 45). More detailed racial and ethnic background of the respondents is not available.

### Measures

#### Reported Closeness, Perceived Impact, and Frequency of Contact

We used the closeness and impact items proposed by Cerel and colleagues (2014) and recently validated as the Suicide Exposure Experience Screener (SEES; Maple et al., 2022). For these questions, participants were instructed to think about the person whose suicide death was most impactful on them. For closeness, participants were asked to indicate how close they were to that person, with five response options ranging from *not close* (1) to *very close* (5). For impact, they were asked to rank the impact of that death by responding to the prompt, “The death …” using five response options: *had little effect on my life* (1), *had somewhat of an effect on me but did not disrupt my life* (2), *disrupted my life for a short time* (3), *disrupted my life in a significant or devastating way, but I no longer feel that way* (4), and *had a devastating effect on me that I still feel* (5). Finally, participants were asked to indicate how frequently they were in contact with the person who died by suicide in the six months prior to the death using six response options: *daily* (6), *every few days* (5), *weekly* (4), *every few weeks* (3), *monthly* (2), and *infrequently* (1).

#### Distress

The Kessler Psychological Distress Scale (K10) (Kessler et al., 2002) includes 10 Likert-type items (i.e., tiredness, nervousness, hopelessness) which participants rate in terms of how often they experienced the issue in the last 30 days. Response options include *none of the time* (1), *a little of the time* (2), *some of the time* (3), *most of the time* (4), and *all of the time* (5). Scores on the K10 range from 10 to 50 with higher scores indicating greater levels of distress. Cronbach’s alpha was .942 for this sample, indicating an acceptable level of reliability.

#### Open-Ended Prompt

The full survey concluded with a prompt for participants to share any further experiences with an open-text response option. Nearly half (48%; *n* = 50) of the subsample extracted for the present study utilized this option to further describe their experience and are the main focus of analysis.

### Data Analysis

Univariate and bivariate analyses were performed with SPSS 24 to describe sample demographics and the nature and impact of exposure on the sample of 104 participants (high impact/low closeness sample). For open-ended responses (*n* = 50), we used inductive thematic analysis (Braun & Clarke, 2021). All authors reviewed the data and inductively created initial themes. After initial thematic identification, authors reviewed similarities and differences. First author (RS) created the final themes and codes, integrating themes identified by all authors.

## Descriptive Findings

Table 1 provides a summary of descriptive findings for both the full high impact/low closeness sample (*n* = 104) and the subsample who provided open-ended prompts (*n* = 50). Here, we report on the full sample (*n* = 104). The majority of participants reported knowing someone who attempted suicide (93.3%; *n* = 97). On average, participants reported 6.56 total attempt exposures and 1.67 attempt exposures with a person they described as close. One-fifth (20.2%; *n* = 21) reported no close suicide attempt exposures. The relationship to the person whose suicide attempt was most impactful was almost evenly split between kin (44.2%; *n* = 46) and non-kin (42.3%; *n* = 44), and 3.8% (*n* = 4) reported their own suicide attempt as the most impactful.

**Table 1.** Nature of Exposure to Suicide Attempts and Deaths, Psychological Distress, Perceived Impact, and Reported Closeness.

Characteristic	% (*N*) Out of 50	% (*N*) Out of 104
Type of exposure		
Suicide attempt exposure only	0% (0)	0% (0)
Suicide death exposure only	4.0% (2)	6.7% (7)
Suicide death and attempt exposure	96.0% (48)	93.3% (97)
Attempt exposures	M = 6.52 (SD = 14.67) Range = 1–100	M = 6.56 (SD = 14.33)Range = 1–100
Close attempt exposures	M = 1.57 (SD = 1.38) Range = 0–6	M = 1.67 (SD = 1.62) Range = 0–100
0	20.0% (10)	20.2% (21)
1	34.0% (17)	31.7% (33)
2	22.0% (11)	21.2% (22)
3 or more	18.0% (9)	19.3% (20)
Relationship to person with most impactful attempt		
Father	8.0% (4)	9.6% (10)
Friend	26.0% (13)	21.2% (22)
Mother/mother-in-law	8.0% (4)	6.8% (7)
Brother/brother-in-law	10.0% (5)	5.8% (6)
Cousin	0% (0)	5.8% (6)
Relationship type to person with most impactful attempt		
Kin	38.0% (19)	44.2% (46)
Non-kin	50.0% (25)	42.3% (44)
Self	6.0% (3)	3.8% (4)
Death exposures	M = 6.30 (SD = 15.35) Range = 1–100	M = 4.64 (SD = 10.86) Range = 1–100
Close death exposures	M = 1.26 (SD = 1.74) Range = 0–10	M = 1.0 (SD = 1.36) Range = 0–10
0	42.0% (21)	43.3% (45)
1	26.0% (13)	32.7% (34)
2	14.0% (7)	13.5% (14)
3 or more	18.0% (9)	10.6% (11)
Relationship to deceased		
Acquaintance	18.0% (9)	9.6% (10)
Other non-family	16.0% (8)	11.5% (12)
Friend of a friend	14.0% (7)	9.6% (10)
Father	10.0% (5)	10.6% (11)
Friend	8.0% (4)	16.3% (17)
Relationship type to deceased		
Kin	32.0% (16)	40.4% (42)
Non-kin	66.0% (33)	58.7% (61)
Reported closeness to deceased	M = 1.46 (SD = .50)	M = 1.56 (SD = .50)
Not close (1)	54.0% (27)	44.2% (46)
A bit close (2)	46.0% (23)	55.8% (58)
Perceived impact of death	M = 4.48 (SD = .50)	M = 4.51 (SD = .50)
The death disrupted my life in a significant or devastating way, but I no longer feel that way (4)	52.0% (26)	49.0% (51)
The death had a significant or devastating effect on me that I still feel (5)	48.0% (24)	51.0% (53)
Frequency of contact prior to death	M = 1.82 (SD = 1.68)	M = 1.69 (SD = 1.38)
Infrequently (1)	78.0% (39)	73.1% (76)
Monthly (2)	0% (0)	6.7% (7)
Every few weeks (3)	2.0% (1)	6.7% (7)
Weekly (4)	2.0% (1)	1.9% (2)
Every few days (5)	10.0% (5)	6.7% (7)
Daily (6)	6.0% (3)	2.9% (3)
Time since most impactful death (years)	12.10 (SD = 13.41)	10.03 (SD = 11.59)
Kessler psychological distress scale (K10) total	M = 22.63 (SD = 9.12)	M = 22.09 (SD = 9.13)
Low (10–15)	30.0% (15)	33.7% (35)
Moderate (16–21)	20.0% (10)	18.3% (19)
High (22–29)	24.0% (12)	24.0% (25)
Very high (30–50)	24.0% (12)	23.1% (24)

*Note.* Full sample (*n* = 104) includes all participants in the high impact/low closeness group. The smaller sample (*n* = 50) only includes participants who provided a qualitative response to the open ended question in the survey.

All participants reported suicide death exposure which was the inclusion criteria for the survey, with an average of 4.64 suicide death exposures and 1.00 close death exposures. Notably, 43.3% (*n* = 45) reported no close death exposures. The time since the most impactful death was 10.03 years on average. Over half (58.7%; *n* = 61) reported a non-kin relationship to the person whose suicide death was most impactful. The five most commonly reported relationship types accounting for 57.6% of all participants included friend (16.3%; *n* = 17), other non-family (11.5%; *n* = 12), father (10.6%; *n* = 11), acquaintance (9.6%; *n* = 10), and friend of a friend (9.6%; *n* = 10). Continuing to focus on the suicide death that was most impactful, participants reported an average closeness of 1.56, with 44.2% (*n* = 46) of participants describing the relationship as “not close” (score of 1) and 55.8% (*n* = 58) as “a bit close” (score of 2). The average perceived impact was 4.51. Nearly half (49.0%; *n* = 51) reported the death’s impact as “the death disrupted my life in a significant or devastating way, but I no longer feel that way” (score of 4), and 51.0% (*n* = 53) indicated “the death had a significant or devastating effect on me that I still feel” (score of 5).

On average, participants reported low frequency of contact with the deceased prior to the death, with the majority (73.1%; *n* = 76) reporting contact as “infrequently.” Only 2.9% (*n* = 3) reported daily contact. Participants reported an average of 22.09 (SD = 9.13) on the K10 measure of global distress, which falls within the category of high distress. Levels of distress were significantly correlated with perceived impact (*r* = .38; *p* = .008), suggesting that high levels of perceived impact were associated with general distress.

## Qualitative Findings

We deductively coded for content related to the constructs of closeness and impact, and then inductively coded beyond this. We present both thematic areas with detailed information about component codes. Participants own words are used verbatim (italics) to provide examples of the themes, with the reported closeness, perceived impact, and relationship to the person who died provided as context.

## Impact of Suicide Death

In this section, we describe five themes that illuminate the experience of impact. As we focused in on the ways in which people described their relationship to the person who died, we noticed a pattern of participants providing justification of the impact they experienced following the exposure, at times even questioning the legitimacy of their response and their “right” to their reaction. Tentative or explanatory language framed their discussion of the relationship and their response to the death. Given the broad nature of the question posed in the survey, the responses varied significantly. While some participants spoke about the nature of the impact resulting from exposure to suicide and specific forms of impact experienced (e.g., hypervigilance, their own suicidal thinking, etc.), others spoke more directly about the circumstances that contributed to, influenced, or complicated the impact resulting from the exposure. Others described the circumstances they believed contributed to the death. It was difficult to disentangle impact and circumstances entirely, and we instead present them as parts of a cohesive whole.

### Theme 1: Impact Through Society and Systemic Circumstances: (Lack of) Recognition, Response, and Responsibility

This theme explores how impact for some respondents was mediated by the response from others, such as friends and family, people in the workplace, and society more broadly. The distinction between impact and the circumstances influencing impact becomes clear in this theme, as participants descried how it was not about the death alone but rather the context in which the death occurred and the stigma surrounding mental ill-health and suicide that impacted them the most. The first subtheme illuminates the experience of and impact resulting from the suicide death exposure not being acknowledged or validated by others.

#### “We are Human Too:” Overlooked and Not Recognized as Being Impacted

Participants described experiences of being forgotten or not recognized and validated following the death. For some this contributed to confusion and questioning themselves, their experience, and their response. One police officer conveyed how first responders are often overlooked when thinking about the impacts of suicide: “*I hope you [researchers] don’t forget the emergency services, because we are the ones who deal with the families initially and quite often on an ongoing basis, sometimes for years afterwards, and we are significantly impacted as well”* [No relationship, Not close, Impact of 5]*.* His outright instruction not to forget implies an experience of being omitted or looked over, of not being recognized. After several sentences describing the extent of his exposure, he said it “*has an ongoing and lasting effect to cops as well, and has contributed a great deal to why I am no longer a [Location] police officer and have been diagnosed with PTSD with all of the usual symptoms and signs.*” His additional testimony highlighted how lack of recognition for the experience can contribute to downplaying the impact or feeling compelled to justify the response.

Another first responder—a firefighter—conveyed a similar message of being overlooked, reminding others of their humanity and the impact of their work: “*Suicide affects a lot of the Triple 0 network who attend suicides on a daily basis, we are the ones that witness the tragic circumstances that most people would dread. We are human too and we get affected by what we do”* [Member of general public, Not close, Impact of 5]. Here, the respondent’s references to being “human”, of being “the ones,” and acknowledgment that they “too” are affected suggest a desire to be seen and imply a lack of recognition on some level.

#### “What was Making Me Angry was all of the Social Context:” Stigma and Responses to Suicide and Mental Health Within Society and Systems of Care

The second subtheme—stigma— explores the ways in which recognition from others can create further harm through stigmatizing attitudes and responses. While the thread of stigma weaves throughout other thematic areas, we included stigma as a separate subtheme given its relative significance in the response participants received from others. Participants identified society’s general attitude towards suicide and mental health as actively detrimental and harmful. Stigma was often directly connected to the experience of silence, and some participants implicated stigma in the inability to talk about the experience openly and receive necessary support. Participants noted that stigma and societal responses made it difficult to access support.

One individual, whose father died by suicide, described how the legacy of suicide in her family cast a shadow through which others saw her: *“On the rare (1-2) times that I sought help from a counsellor - I felt that they focused too much on the effect on me of my father’s death when what was making me angry, frustrated & a bit ratty, was all of the social context - cultural beliefs, attitudes, stigma etc & untrained (as well as trained) people’s views & expectations that I was genetically tainted/pre-determined to have a mental illness/underprivileged existence. That was ignorant & wrong”* [Not close, Impact of 5]. Others were unable to see past her family’s history of suicide when they sought out support. Another participant commented on the response to a suicide in the workplace and the subsequent lack of support for staff in systems of care. The severity of the shared trauma was dismissed by management: “*The lack of empathy towards staff involved by senior management was appalling. This has had an enormous impact. The ability to discuss the event and need to implement change was severely curtailed. This added to staff distress”* [Patient, Not close, Impact of 5]. This response speaks to organizational silencing, a specific circumstance that influenced the reported felt impact.

#### “It’s Hard to Put into Words:” Silenced and Silencing

Silence was more directly named by some participants. Stigma, silencing, and a lack of recognition were implicated in availability of and access to support. Stigma surrounding suicide contributes to the experience of being silenced and difficulties accessing support, which intensified the impact of the death. One man described stigma associated with the loss impacts the ability to share openly and receive support needed: “*It’s the silence around the Suicide I believe hurts people the most. Not only are a family grieving, but they don’t know where to turn. It hurts beyond belief, things are changing slowly but I hope there will be positive changes in my lifetime*” [Family friend, A bit close, Impact of 5]. For some participants, silencing occurred due to temporal expectations, particularly a perceived timeline for grief and when people should “move on,” as noted by one parent who felt left behind in their grief while everyone else moved on: “*Still too hard for me & most people don’t want to talk about [my son]’s death or have forgotten now. As his birthday was yesterday - I feel very sad.*” [A bit close, Impact of 5].

Some participants described a form of silencing that resulted from difficulties articulating the experience to others. A lack of language to talk about the experience may be connected to stigma or simply not having recognition of the experience from others. A train driver described the significant impact of her exposure: “*As a train driver, it has effected* [sic] *me at work and in many life situations. The impact has created a reaction to certain physical movements that people make when I am driving a car, a train, a truck or even riding a bike. It has made me super sensitive to mitigating danger to individuals. It has effected* [sic] *me emotionally but it’s hard to put in words*” [Pedestrian, Not close, Impact of 4]. A lack of recognition and recognition through the lens of stigma contributed to silencing and an inability to talk about the experience, included as a separate subtheme. While the reasons differed, participants described situations where they were not recognized as being impacted by the exposure or where opportunities to talk about the experience were curtailed.

### Theme 2: Impact Through History and Repeated Exposure: The Accumulative Effect

This theme refers to repeated exposure within the family or workplace and how the legacy or history of suicide exposure follows participants over long periods of time. This type of impact was more diffuse; rather than being mediated by external circumstances (as in theme 1), impact through repeated exposure or family history seeped into various aspects of the participants’ lives. Cumulative exposure typically occurred in two ways: More commonly, responses spoke to repetitive exposure to suicide through work, while others were exposed via family history and/or legacy, as some participants commented on multiple suicide deaths occurring within the family or community.

#### “It Runs in the Family:” Family History or Legacy

This category includes responses that speak to the legacy of suicide and its impact on multiple generations within the family. Shifts in family dynamics related to suicide reflected a normalization of suicide through repeated exposure. One woman who experienced repeated suicide deaths across several generations and whose father died explained suicide “*runs in my family*” [Not close, Impact of 5], suggesting a sense of inevitability and resignation to the notion of suicide as an immutable fact and biological inevitability. For another woman, family secrecy of her father’s suicide led her to feel “*disappointed and angry*” with other family members, especially as she “*had thoughts of suicide myself*” [Not Close, Impact of 4]. Some participants recognized unhealthy strategies they adopted for coping with suicide in the family. One participant described how her “*falling into an unsuitable relationship with my mother’s death”* led to unhealthy relationships with romantic partners and an inability “*to discuss my mother’s death with anyone*” due to adopted patterns of family secrecy, eventually leading to passing “*intergenerational effects of mental illness and suicide*” onto her two children [A bit close, Impact of 4].

Participants also described impact as a systemic process felt within the family system. Although one participant never met her deceased father, the impact of his death by suicide was still felt at an individual level and within the family: “*Suicide has long lasting impacts on the families of those affected for many years after the event . . . I grew up never knowing my father but the cause of death was a great distress to my family and to me for years, we have carried this burden. It’s always a difficult conversation to have when meeting new people who ask about my father.”* For this person, witnessing other’s grief and response to the death was also traumatizing: *“My mother blames herself for not being able to save him in his last attempt . . . it is a distressing subject that still causes emotional outpourings at every anniversary”* [Not close, Impact of 4]. Although not a common pattern across most participants, one Aboriginal woman whose family members “*have lost a young Aboriginal male to suicide in every generation since colonisation/invasion”* yet consequently created a positive shift in their family dynamic. She described the effects as “*a process of rebuilding family relationships over the last 60 years in my family”* through which the losses have *“opened up a space for family to connect, I feel guilty because of this association between the family’s loss and my personal gain”* [Not close, Impact of 5].

#### “We Attend Suicides on a Daily Basis and it Never Gets Any Easier:” Cumulative Exposure

Participants who described cumulative exposure via the workplace focused on the impact of responding to the traumatic death scene and being responsible for relaying the news to loved ones. First responders commonly described PTSD as an outcome of repeated exposure to suicide on the job, and for some, it contributed to time off work. A train driver shared, *“I also experienced a man [description of method] while I was. . . next to him. This had less impact on me but did have some emotional impact”* [Pedestrian, Not close, Impact of 4]. Repeat exposure to suicide deaths for one frontline responder caused PTSD severe enough to cause disruption to his work. He shared that he “*attended numerous suicides in my role over 21 years”* and *“was medically discharged from a job I loved due to PTSD Post Traumatic Stress Disorder directly related to my work as a firefighter”* [Member of general public, Not close, Impact of 5].

One police officer illustrated how thoroughly frontline workers can be exposed to be suicide death and how deeply they can be impacted as a result. Notably, the police officer did not describe impact from the suicide scenes themselves but rather the weight of being the one to convey devastating news to families over and over:

“I couldn’t count the number of times I have knocked on a door to tell a family member, a mum or dad or husband or wife or kids, the number of times I have had to break their hearts. It’s a really difficult thing to do... and it is a task that NEVER gets any easier, because if it was easy we would have no compassion or empathy left. Over and over again, strangers and then sometimes people you know, breaking their hearts and ruining their lives with one short conversation, one knock on the door in the middle of the night. Because that’s what we do. We knock on a door and we completely change a person’s life in one or two sentences. . . They don’t show that stuff on TV...not really. And when you do that day after day, year after year, it affects you. How couldn’t it? No-one should have to do that sort of thing for as long as we do. . . To me it was always like concentric ripples, flowing out from one act, and police are often just as affected” [No relationship, Not close, Impact of 5].

Impact through repeated exposure to suicide within the family or workplace seems to be a heterogeneous experience. The source of exposure in these instances—work or family—mattered, and had clear implications for how participants interpreted or experienced accumulative impact.

### Theme 3: Impact Through Other People: The Ripple Effect

In this thematic area, we identified subthemes that reflect the ripple effect of suicide beyond individual participants to families, groups, and communities. Participants often recognized they were not close to the person who died but still were grieving through others or were concerned with the impact for others who were close to the person who died. The impact of these suicide deaths were mediated by other people.

#### “It Weighs on My Mind Constantly:” Fear of Transmission and Concern for More Direct Impact

The emotional tenor of fear resonated in participant responses, exhibiting as fear of others using suicide as an option, fear of being more directly impacted by suicide and recognition of the tragedy of direct impact, and vigilance as a response to fear and an effort to prevent transmission. This subtheme is closely connected to family history and legacy, as it is often through family history that participants were first exposed to suicide. For some participants, the meaning of suicide as family legacy was centered on fear. At times, this fear of transmission motivated a desire to change the family story about suicide. One participant shared, “*Given my family’s history of suicide in the young Aboriginal male per generation, I worry for my own teenage child. He has had adverse childhood experiences, I have made every effort to keep him engaged and connected socially . . .I worry that I haven’t been able to support him developing sufficient resilience to persist in this world and he might be next. It weighs on my mind constantly”* [Cousin’s child, Not close, Impact of 5]. Another person described the impact as “*It’s also the grief for the family and the fear for my own son for a period of time while he was a teenager”* [Cousin, Not close, Impact of 5].

Other participants shared their experience not of fear of transmission but of witnessing transmission in real time, especially in a small community. One participant described a community wide desensitization to and subsequent transmission of suicide: “*The impact upon the young children/people in my location and how they quickly proclaim, when in a tough situation stating that they should kill themselves just like X did. Then 6 month[s] later one of X parents committed suicide ... I felt polarised by this and recalled how devastated this family had become since the death of their Matriarch and how I have been a witness to their collective implosion”* [Niece, A bit close, Impact of 4]. These responses reflect how some participants felt helpless and perhaps frozen by fear, others were galvanized to action.

#### “It has a Worrying Effect Causing Concern for People Closer to the Deceased:” Concern for Grief and Distress Experienced by Others

Responses in this subtheme speak to bearing witness to and holding concern for the impact of the death on people with a direct and close connection to the person who died. Here closeness and impact intersect, as participants describe that the impact of the death was mediated through those who are closely connected to the person who died, with concern for how other people cope with the loss and empathy for those left behind. One person felt impacted because she empathized for the people who were witnesses to the death and the loved ones who survive the deceased: “*I always wonder how so many people that where witness to such a horrible situation were able to move on from it … .how much it affected there* [sic] *life and wellbeing*” [My friend’s husband, A bit close, Impact of 4]. Another respondent was impacted by her concern for the well-being of their granddaughter following the death: “*The effect on me and my life has been through the devastating and probably lifelong effect it has had on my granddaughter, who is now suffering from PTSD and is having treatment at this time. It has effected* [sic] *us as a whole family and remains to do so”* [Granddaughter’s boyfriend, A bit close, Impact of 5].

At times, participants offered recognition and acknowledgement that they were not close to the person who died. For example, one woman shared, “*We were impacted by the distress it caused the parents who were very close friends, not because we were close to the person who died so much”* [Friend’s son, Not close, Impact of 4]. For one elderly man whose neighbour died by suicide, his daughter-in-law worried about the fallout on his mental health: “*They were best friends for over 40 years and Poppa now has none with whom he had daily contact. He is 88 and increasingly frail and I worry about his mental health. (He was the person who found the deceased.)”* She went on to say, “*Similarly a recent suicide in [Location] had impacted directly at least two people in [Location] that I know who had worked closely with the deceased and I was the person who had to tell them of his death. This is not fun”* [Acquaintance, Not close, Impact of 4].

### Theme 4: Impact as Motivator for Reflection or Change: Reconsideration and Review

This theme includes responses that speak to existential questioning and reflections about life, death, and meaning. For some people, the challenge of the death prompted a process of meaning making/reconstruction and personal growth, and their reflection, questioning, and exploration contributed to active and positive change. For others, reflection and questioning were connected to a deep sense of empathy and sorrow for the pain of those who died by suicide, which is in contrast to the previous theme where the focus of concern and empathy is those who were more closely connected to the person who died. This theme reflects a more direct impact, although responses center on both process and outcome related to impact.

#### “It Changed My Life:” Positive Change or Growth in Outlook or Perception

Growth and positive change were prompted for some by reflections on suicide and death broadly. For some participants, this change was reflected in their attitude about living and connection to life. For others, exposure to suicide shaped their professional trajectory, motivating their work in the area of suicide prevention. Although many participants expressed how suicide exposure opened the door to their own suicidal thinking, one participant described how exposure to the suicide death of a peripheral connection prevented their own suicide. They shared the insight they gained into their own struggles through the death of another person and the experiences of those left behind:

“I went to a party at the house of my friend who[se] brother had died for 'one last night of partying'. His mother was there. She talked to a group if [sic] us about [her son] until the early hours of the morning. I stayed all night knowing that it would keep me alive. A part if [sic] me just wouldn't give in. That night has changed my life. His mum said something that I think of often, 'As devastating as it is for a parent to hear that their child wants to kill themselves, it is nothing compared to losing that child to suicide.' That night changed me forever” [Brother of my boyfriend at the time, A bit close, Impact of 5].

Family history or legacy here can be viewed as a prompt for meaningful change and a motivation to work in the field of suicide. A family exposure to suicide and the questions surrounding the experience pushed one participant to pursue a career in suicidology: “*I was totally confused as to why my Uncle died by his own hand . . . because of my experience I went on to obtain an extra qualification [in suicidology]. I have spent 12 years as a telephone counsellor with one organization and spent 4 years with another organization as a Trauma Response worker. All of this generated out of my experience when I was younger”* [A bit close, Impact of 4].

Following her father’s death, another participant’s family was fearful of suicide transmission, resulting in vigilance over a family member, which is similar to the quote in the past theme. Here however, communication and strengthening of familial bonds contributed to assuaging these fears, and efforts to disrupt family patterns and fear of transmission created motivation to work in suicide prevention: “*I found out my father’s death was in my late 20s and at that time I was unaware that he died of suicide. It was a time that I had thoughts of suicide myself. I have a twin sister who has been unwell (depression) over the years and has been suicidal and all of this makes us as family extra vigilant. However, I believe that over the years my sisters and I have spoken about many things and this has helped us enormously. I now work in the area and am eager for the myths about suicide and for the conversations to continue”* [Not close, Impact of 4].

#### “I Don’t Even Know Why I’m Grieving Somebody I Don’t Know:” Compassion for Life Lost and Empathy for Personal Struggles

Although they were not connected through closeness, several participants reported a deep compassion for the suffering of the person who died and the life that was lost. This theme also expresses concern for the experience of others, yet it is not about the person who is grieving the loss (as in the concern for others theme above). Instead, this concern is focused on the person who died. One participant described questioning her deep experience of grief: “*I have grieved for a child I don’t know, never met but she was my 17 year old daughter’s best friend …. she was 16 when she tragically took her life, her mother found her [description of methods] & I have had significant trouble coming to terms with her death …. I don’t even know why I’m grieving for somebody I don’t know”* [Not close, Impact of 5]. For another, the death of a friend’s relative led them to think about the isolation of those suffering: “*Just how devastating suicide is when the person succeeds and mostly without letting those around them know the bad place they are in and why they intend to take their life”* [A bit close, Impact of 4].

#### “I didn’t Know They Were Struggling, but I Feel Like I Failed Them:” Sense of Responsibility and Guilt

For some participants, deep empathy and compassion for the struggles of others also prompted a sense of responsibility and guilt. At times, a sense of responsibility arose from recognition of suicide as an internal experience, often impervious to the best efforts of others. While participants questioned what they might have missed, they also noted that the person concealed their pain so that others were not aware of the depth and magnitude of their struggles. Participants recognize that the death was not their fault, yet still reported guilt and questioning whether they could have done something to prevent the death.

Participants who carried a sense of responsibility and guilt often described this obligation arising from their connection to the community in which they were a leader. For example, one person described a peer’s death from a class where the respondent was a leader and shared “*This had a massive impact on me and led me towards self harm and contemplating suicide”* [Not close, Impact of 4]. After the suicide death of an acquaintance, one individual felt surprised at how imperceptible their struggle seemed and guilty that they did not notice anything was wrong:

Every year my husband and I organise a social weekend at the snow from people we know through the kayaking community (High school and club mostly). This person came 3 years in a row. He was handsome, with a good job, quiet, great with his kids (3 young ones), funny and at no time did we ever thought he was even depressed. 3 years ago, he came with his kids again, enjoyed the weekend, joked with us, pretended to be OK and even made plans to join the committee. 3 days later he killed himself at home. I was angry with him for doing that to his kids and wife (whom I had never met). I was shocked as I did not know any of his struggle. I was profoundly sad that I did not pick it or could help and that someone could feel so much pain that needed to end their life. I still have vivid memories, every time I go back to the snow, of the spot where I last saw him and said good bye to him. It still makes me cry when I think about it.” [A bit close, Impact of 4].

A sense of responsibility was often felt at the individual level, though frustration with ineffectual systems connected to a sense of responsibility for some participants. One participant described feeling partly responsible for the death by suicide of three youths for whom she advocated. This responsibility was connected to frustration with unresponsive systems, particularly the local government: *“The [Location] government closed down the extended treatment facility and myself and others in the community told them that would result in suicides, as did adolescent clinicians. Our warnings were ignored. I still feel like I failed them in some way, wondering if there was more I could have done”* [Son’s friend, Not close, Impact of 5].

### Theme 5: “I’ve had My Own Journey With Thoughts of Suicide:” Impact Through Shared Resonance and Identifying Self in the Other

Participants talked about their own experiences of ideations or attempts, and those who did often mentioned a connection between the suicide exposure and personal thoughts of suicide/suicide attempt. Some participants reported that they were experiencing suicidal thoughts or behaviours at the time of the exposure and felt a sense of identification with the person who died. For others, the exposure created a new pathway to suicidality that did not otherwise exist. One participant who previously experienced ideation sympathized with her friend who lost someone close to her: “*I wasn’t close at all to the girl who committed suicide. But she was my best friend’s best friend, so it hit home. At the time I had attempted suicide before. When this happened I was devastated for her and my best friend, I truly saw the effect it had on her friends and family and people who didn’t even know her. Including myself. We had spoken a few times before. It made me want to get better. . .It affected my entire outlook on life, even though I didn’t know her very well”* [Not close, Impact of 5]. Another participant shared, “*I knew her through school and I was suicidal at the time she died, I only ever saw her in class and we had mutual friends but her death shattered me. Being young it made me realise suicide works”* [Not close, impact of 4].

Family history of suicide was at times connected to personal thoughts of suicide. For one participant, suicide was perceived differently after her father’s death: “*I’ve attempted suicide many times since my father’s suicide. I romanticized suicide a lot in my teens and early twenties. I still continue to think about my death every day though I don’t think about suicide anymore*” [A bit close, Impact of 4]. An extensive family history with suicide led another individual to conceal their own personal experiences and experience a great deal of shame before she was able to seek help: “*I still think about my cousin everyday. . . She attempted suicide so many times over 20 years that we lost count. When she died it was like the weight of waiting (for her to suicide) had been lifted from the family. At the same time, a concrete boulder of grief descended on my shoulders. She was finally gone and that grief was unbearable. I had my own suicide journey and admitted myself to hospital twice. On my second admission I ‘practiced’ my method, but didn’t tell anyone about it. I confessed to my psychiatrist 2 years later and got the support I needed*” [Not close, Impact of 4]. The respondent’s language of “confessing” suicidal behaviours to others reflects the stigma commonly associated with suicide.

## Nature of the Connection to the Person Who Died

**Figure 2.**
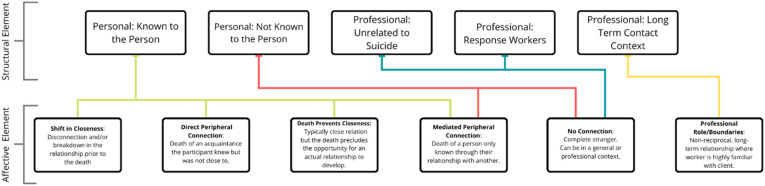
Structural and affective elements of closeness.

We identified a pattern where participants provided more detail about the nature of their connection to the person who died or offered explanation for the relationship with the deceased, at times commenting directly on the closeness—or lack of closeness—they had with the deceased. We organized the responses around two related elements of closeness: (a) a structural element, and (b) an affective element. While the themes pertaining to impact provide insight into what the experience of being highly impacted is like for participants, the closeness themes provide additional explanation about why these themes might exist. Figure 2 provides a visual representation of the relationships between these two elements.

### Structural Element of Closeness

The structural element of closeness refers to categories used, often implicitly, to describe the type of relationship and the socio-cultural expectations around closeness given the relationship type. Relationship types centered on how the individuals were connected (i.e., personally or professionally) and the extent to which they knew each other. First, some respondents described a personal connection to the deceased, varying widely based on whether they knew the person themselves or merely knew of them. Relationships where individuals knew each other in a personal context included family members, friends, co-workers, and so on. Other respondents described a personal connection without directly knowing the individual who died. Examples could include connections in which individuals know of each other through others, cross paths as strangers, or never had any connection or relationships prior to the death yet have a familiar connection. These could include “best friend’s other best friend,” a “friend’s child,” or a “child’s friend.”

Other respondents described professional relationships that varied based on the degree to which they interacted with the person who died. Some relationships occurred after the pair met in a professional setting over a long-term period of time, such as in mental health treatment settings. These relationships were often nonreciprocal, where one person (e.g., a client) shares more of themselves or is more vulnerable in the relationship than the other (e.g., a therapist). This group often included contexts where one might regularly interact with suicidal individuals and therefore anticipate being exposed to another’s suicide. In contrast, some professional contexts included no previous relationship with the deceased yet also came with an expectation that one could be exposed to suicide death. These contexts included people who engage in front-line emergency response work, such as police officers or firefighters. These individuals frequently respond to the scene of the death and might be responsible for communicating the loss to family members. Finally, some individuals encountered a suicide death in a professional setting that was entirely unexpected. This category could include a professional connection unrelated to death (e.g., teacher-student relationships) but often included professionals whose workplace setting is commonly used as a method for suicide (e.g., train drivers, transit workers).

### Affective Element of Closeness

The affective component of closeness refers to an individual’s perception of closeness, often based on perceptions explained beyond the relational. The affective element of closeness provides insight into the participant’s experience of the relationship, the possible reasons for low closeness, or the meaning ascribed to the relationship.

Some participants described a disconnection to the deceased or a breakdown in the relationship that occurred prior to their death. Examples of this category were often personal relationships where the pair knew each other and previously had close relationships but were not close at the time of the suicide death due to conflict or a prior separation. For example, one woman described the relationship breakdown with her brother due to her efforts to seek help for his suicidality, which ultimately led to him refusing to have contact with her, yet his death impacted her greatly because she had tried to advocate for him and received family rebuke for it. Other participants described a type of relationship that would typically be close based on the structural connection (e.g., parent-child) but circumstances precluded the opportunity for a physical, in-person relationship and/or attachment to develop. For example, respondents referred to the death of a parent or grandparent when they were very young or before they were born, preventing the development of a personal relationship.

Some participants described a direct but peripheral connection to the deceased, which included people who the respondent knew directly but with whom they were never close (e.g., an acquaintance, a classmate, a neighbor). For example, one respondent described a peer’s suicide who was in a grade below her but with whom she shared a class: “*I felt a strong sense of responsibility as I was a leader in the class and I did not see he was struggling*.” Other participants described mediated peripheral connections, in which they were never close to the person who died but spoke about a distal link where the connection was through another person. At times, these respondents described their impact of the death being as a result of the impact on the person more closely connected to the death. Participants in this category often acknowledged their lack of closeness and made an effort to explain their response to the death, with recognition that they or others may see this impact as incongruent with the nature of the relationships to the person who died. Alternatively, some responses focused on describing the experience (e.g., what led to the death, how the death occurred, how others coped) rather than the impact experienced by the respondent. Finally, for those with professional long-term connections, duty of care resulting from their professional role influenced their perceptions of closeness and the death’s impact. These examples reflected non-reciprocal closeness, as with mental health providers who know intimate details about a person’s life without sharing the same level of detail about their own. Professionals with short-term connections or in settings unrelated to suicide provided responses that reflect circumstances where they were the first to respond to the scene of the suicide death, either due to their work role as a first responder or due to the death occurring in public place where members of the general public witnessed or responded to the death (at times known as a zero responder). For example, one respondent shared “*my husband and I were the first on the scene and we had to try and revive this person.*” The distress from this event had a great level of impact despite having no closeness to the person who died.

## Discussion

Through exploration of qualitative comments provided by people experiencing high impact resulting from suicide death exposure where they had a low level of closeness to the decedent, this study contributes to expanding and reconceptualizing our understanding of impact following exposure to suicide. The current conceptualization of the Continuum Model (Cerel et al., 2014) suggests a range of responses moving from less impact to more impact corresponding with increasing closeness which assumes attachment with exposure on one end and bereavement on the other. The findings of this analysis suggest that there are other forms of substantial and life-altering impact beyond the loss of an attachment that can be explained through a bereavement lens. Some of the ways in which participants described impact here align with common reactions reported by friends and family, yet there are clear differences. Importantly, experiences aligned with what we might consider traditional bereavement were rare. Rather, participants described common reactions (e.g. stigma, silence, guilt, fear of transmission) complicated by a lack of validation and acknowledgement from others. This adds further layers of complexity that have yet to be reported in the literature.

Our results offer insight into the complex nature of closeness and the various factors that affect the perception and experience of closeness, suggesting that it is not merely how people are connected to individuals who die by suicide, but also how they experience the relationship (Cerel & Sanford, 2018). While each relationship is unique, high closeness is homogenous in the sense that it reflects an attachment relationship. This is not true for low closeness, and our results offer insights that further our understanding of the substantially varied and diverse reasons for and meanings of low closeness. For example, the complex nature of closeness is highlighted in situations where death precluded the opportunity for closeness with someone who would typically be a close kinship tie. Such situations may reflect a type of ambiguous loss (Boss, 2006), where it is not the actual relationship the person grieves, but rather the loss of a potential relationship or what could have/should have been. This parallels typical bereavement, where people grieve not only the immediate loss of the person in their daily life but also their imagined or planned future together.

The complexities of closeness are also reflected in comments indicating a shift in relational dynamics prior to the death. Although shifts in closeness due to conflict and disconnection prior to the death did not occur commonly in this sample, literature points to the significance of relationship quality as a factor for the bereavement experience (Bottomley et al., 2019; Sekowski & Prigerson, 2022). Relationship breakdown is a common precedent for suicide (Stone et al., 2018), which suggests that relational conflict or shifts could be common for people exposed to suicide loss. Experiencing relational conflict and disconnection prior to the death may contribute to complicated grief and grief-related psychopathology for those left behind. Current measures of closeness also do not account for changes in relationship closeness over time. Closeness may not be a static experience, and instead may fluctuate over time.

Thinking beyond kinship connections, friends are often the most common relationship reflected in large-scale and representative research on suicide exposure (Feigelman et al., 2018; Maple et al., 2019; Maple & Sanford, 2019), yet they are often not reflected in the literature on suicide bereavement (Maple et al., 2014) or bereavement generally (Johnsen & Tømmeraas, 2022). The peripheral connections identified in this study reflect a group even less likely to be studied than friends. While often not reflected in the literature (Fingerman & Hay, 2002), peripheral connections (or “ties” as used by Fingerman, 2009), serve a variety of functions in our daily lives, such as social integration, information and resource sharing, emergency support, and assistance with daily tasks (Fingerman, 2009). Their absence in our lives may be felt differently than the absence of intimate connections, though this may not reflect a loss that is less-than or insignificant.

The professional side of closeness illustrates points of overlap and divergence among different occupational groups commonly exposed to suicide in their work. Suicide is generally considered an occupational hazard for people working in mental health (Chemtob et al., 1989), which may contribute to the way in which worker response is minimized or dismissed. As mental health workers are expected to encounter and even be prepared for exposure to suicide deaths, the impact resulting from such exposure is often not considered. Likewise, although exposure to suicide is routine in the work of first responders, their experiences are rarely reflected in the suicide exposure literature (Kimbrel et al., 2016; Koch, 2010; Nelson et al., 2020). Public safety personnel in this study pointed clearly to their experience of impact being overlooked. As the field of suicide exposure has been dominated by a bereavement focus, people without an attachment relationship to the decedent are assumed to be less impacted or simply have not been the focus of investigation into their occupational and personal experiences.

Literature focused on the grief experiences of friends offers an important point of connection for interpreting our findings (e.g., Bartik et al., 2013a, 2013b, 2015; Johnsen & Tømmeraas, 2022). Friends, particularly youth and young adults, are frequently overlooked as grievers, with little attention paid to their experience, despite the fact that friend is often the most common relationship reported in suicide exposure research. In their study of young adults grieving a friend, Johnsen & Tømmeraas (2022) found that “In some ways the friends felt forgotten, and not entitled to grieve in the same way as family or an ‘inner circle’ of those affected...In addition to being forgotten or not receiving help, it seems like some of the friends don’t recognize themselves as being bereaved, even though they clearly are suffering and grieving the loss of their friend” (p. 6). This lack of recognition was articulated in the experiences of first responders in this study. Disenfranchised grief offers one lens through which to understand this experience (Doka, 2002). However, the comments from participants do not reflect grief, per se, but rather other forms of impact. Nevertheless, comments from participants reflect the experience of being disenfranchised from acknowledgement and social validation often important to making sense of experiences.

Our findings suggest that this cumulative exposure further complicates impact. Participants with workplace exposure named PTSD as a form of impact, echoing other research with public safety personnel (Nelson et al., 2020). Further, although repeated exposure to suicide may be common, it is often not acknowledged as potentially traumatic, nor are resources for coping discussed (Nelson et al., 2020). People commonly experience multiple exposures, with some groups more sensitive or vulnerable to the effects of accumulative impact of exposure. Exploring cumulative exposure and the mechanisms that contribute to an accumulative impact is important for future research.

The sense of responsibility and guilt reported by participants warrants further exploration. Guilt and responsibility are common in the suicide bereavement experience, as close friends and family question what they missed or what they could have done to prevent the death (Jordan & McIntosh, 2011). However, given that participants in this study did not have a close relationship to the person who died, it is likely that they would not have been close enough to have access to information that would have allowed them to know the person was struggling or to intervene to help. We might consider this an act of sense making, whereby participants attempt to gain a sense of control or power over terrible things that happen unpredictably and unexpectedly. It may also be connected to sociocultural messaging around suicide and suicide prevention, which often emphasizes the importance of noticing warning signs in order to effectively help someone who may be at risk of suicide. Future research may explore how messaging about warning signs potentially reinforces notions of personal responsibility and guilt (Jordan, 2017), especially for those who have completed gatekeeper training.

Exposure to suicide is a noted risk factor for suicidal thoughts and behaviours of kin (Pitman et al., 2014) and non-kin (Maple et al., 2017; Song et al., 2015). Personal experiences of suicidal thoughts and behaviours were common among participants in this study, with nearly 30% (*n* = 15 of 50) reporting their own suicidal thoughts or behaviours in the qualitative responses without any prompting to do so. We are not able to comment on the temporal or directional nature of the relationship between suicide exposure and participant experiences of suicidal thoughts and behaviours, though it seems that some participants identified with the deceased person’s state of mind, despite the lack of closeness in their relationship. How this relates exposure to suicide contagion is beyond the scope of this study but worthy of future investigation.

Overall, the results also encourage a deeper exploration into closeness and relationship quality as a factor influencing perceived impact following exposure to suicide death. Previous research has called for assessing perceptions of closeness beyond categorical definitions of relationship and the kin/non-kin divide (Cerel et al., 2016). However, this study suggests that we need even more nuanced explorations of closeness. For example, one woman in the high impact/low closeness sample lost her son. Her inclusion in this group raises questions about the complexities of closeness, and offers a reminder that the structural nature of the connection between two people does not necessarily determine their affective experience of closeness. This is particularly true in some cultures or communities (e.g., Indigenous communities) where kinship extends beyond blood ties. Similarly, we need to consider situations where a person may be excluded from the family due to conflicts in values or beliefs (e.g., people who are sexual or gender minorities). In order to develop greater understanding of closeness and how it influences the impact of suicide exposure, future research should explore not only the structural elements of closeness (relationship type), but also the affective components of closeness, including perceived closeness (attachment) and the quality of the relationship.

The findings also encourage us to reconsider how high impact has been conflated with bereavement in the existing suicide exposure literature, and in so doing has missed these complex, important experiences beyond bereavement that are important to consider. This study encourages a more nuanced understanding of impact, with attention to the circumstances that contribute to the impact following exposure to suicide. Mediational models that examine the impact of factors identified in this study (e.g., stigma, recognition, social support) and others may help advance our effort to understand who is significantly impacted by suicide exposure and their needs for support.

## Implications

The sample in this study represents a small group of participants from a larger sample (3.5%), though notably these participants experienced high impact from their suicide exposure and would typically be considered bereaved within the Continuum Model. Their experiences, which do not reflect bereavement, require attention as they are incongruent with the current evidence in the emerging suicide exposure field. These participants indicated high levels of impact, with many rating impact as a 5, suggesting they may be in need of support to mitigate potential harms associated with the experience. However, they are unlikely to be identified through traditional means of outreach and services because systems and providers are not expecting them, and thus, services are not designed for them. Further, they may not voluntarily seek support, or believe they are eligible for services, particularly given noted limitations in the support available (Song et al., 2015). Postvention efforts should target those most at risk for significant impact, particularly acquaintances and other infrequently recognized relationships who may be at increased risk for life-altering impact, including their own suicidal thoughts and behaviours (Song et al., 2015). To ameliorate harms associated with suicide exposure, we need to explore services and intervention approaches tailored to unique needs based on relationship closeness and quality with consideration of the ways in which their experiences may be disenfranchised. Dimensions of connection and closeness to the deceased impact the grief experience, and must be addressed appropriately by helping professionals through individualized support (Abossein et al., 2022; Cutrer-Párraga et al., 2022). Past research has established this with bereaved friends, identifying young people who require additional attention and support (Bartik et al., 2015), and this is supported by Johnsen & Tømmeraas (2022) who report implications when these relationships are not recognised. The current study adds further support for this.

For some participants, access to support was curtailed by stigma. Stigma is associated with hesitation or reluctance to openly share about grief and loss experiences (Maple et al., 2014). Participants described that finding good support was important, but that stigma and the lack of openness around suicide meant that they did not know where to turn for help despite best efforts. The results raise questions about the difficulty participants experienced in trying to find the right supports. Specifically, we remain curious about whether difficulty accessing support reflects a lack of fit with the existing supports or logistical barriers (e.g., cost) to accessing appropriate supports that currently exist.

The results of this study provide important instructional information for using the Suicide Exposure Experience Screener (Maple et al., 2022). The validated measure includes two items—the closeness and impact questions used in this study—that would identify people with high impact/low closeness, thus supporting providers to consider the unique and individualized needs of the person.

If we want to take seriously the notion of postvention as suicide prevention (Jordan, 2017; Shneidman, 1972), we need to think beyond bereavement to inform who requires postvention attention. While bereavement may be one outcome of suicide exposure, it is not the only outcome. Bereavement implies that one has lost a significant loved one to death. Examining suicide exposure through the lens of meaning making and narrative approaches (e.g., Miklin et al., 2019), we realize that exposure can shake or shatter global assumptions about the world, our place in the world, our relationships to others, and the foundational beliefs of security, safety, and predictability.

## Limitations

The findings from this study should be examined with its limitations in mind. First, the open-ended question coded in this study was a general question asking respondents to provide any additional information they wanted to share about their experience, and thus those who responded were few in number overall and their experiences may be similar to and different from those who did not respond to this question. Future research should incorporate more nuanced and specific questions to further understand the experiences of people who report high impact following the suicide death of someone with whom they had a low level of closeness. Additionally, the themes we identified are seemingly disconnected from one another, in that it is difficult to find a thread that connects all of the themes. This could be due to the nature of the prompt included in the analysis; the question did not ask participants explicitly to provide additional detail about the impact resulting from the suicide exposure, but rather asked participants to share any other information about the experience that they wanted to share. As the substantive content of the responses varied, the themes did as well.

## Conclusion

Previous research has demonstrated the extensive impact suicide exposure can have, yet has failed to address the nuances of those who feel highly impacted from the death of someone to whom they were not close. Our study exploring participants who identified being highly impacted by the suicide death of a person to whom they were not closely connected illuminates experiences on the margins of suicide exposure. Our findings highlight the need to tailor interventions that acknowledge these nuances and address the uniqueness of individuals’ experiences.
